# The role of the UNITE4TB Young Investigators Group in empowering early career researchers through mentorship, capacity building, and innovation

**DOI:** 10.5588/ijtldopen.25.0651

**Published:** 2026-02-11

**Authors:** A. Vasiliu, F. Saluzzo, J.A. Schildkraut, R. Villar-Hernandez, J.P. Ramos, M. Vieira, G. Thwaites, D.M. Cirillo, C. Lange, R. Duarte

**Affiliations:** 1 Clinical Infectious Diseases Department, Research Center Borstel, Sülfeld, Germany;; 2 Clinical Tuberculosis Unit, German Center for Infection Research (DZIF), Borstel, Germany;; 3 Global TB Program, Department of Pediatrics, Baylor College of Medicine, Houston, TX, USA;; 4 IRCCS San Raffaele Scientific Institute, Milan, Italy;; 5 Radboud University Medical Centre, Department of Pulmonary Disease, Nijmegen, the Netherlands;; 6 Genome Identification Diagnostics (GenID), Strassberg, Germany;; 7 EPIUnit, Instituto de Saúde Pública da Universidade do Porto, Porto, Portugal;; 8 Instituto de Ciências Biomédicas Abel Salazar, Universidade do Porto, Porto, Portugal;; 9 Oxford University Clinical Research Unit, Ho Chi Minh City, Vietnam;; 10 Nuffield Department of Medicine, University of Oxford, Oxford, UK;; 11 Respiratory Medicine and International Health, University of Lübeck, Lübeck, Germany;; 12 Centro de Saúde Pública Dr. Gonçalves Ferreira, Instituto Nacional de Saúde Dr. Ricardo Jorge, Porto, Portugal.

**Keywords:** tuberculosis, mentorship, guidance, early career, young investigators, research consortium

Dear Editor,

Early career researchers (ECRs) play a crucial role in reforming and strengthening research culture and tackling systemic challenges in science.^1^ Research consortia can support ECRs by offering mentorship, skill development, and opportunities for interdisciplinary collaboration, helping them navigate the competitive research landscape and establish successful careers. Networking opportunities provided by consortia further enhance ECRs’ visibility and career prospects.^2^ Through participation in publications, conferences, and collaborative projects, ECRs can build meaningful professional relationships with peers and senior scientists.^3^ Additionally, consortia are crucial in promoting diversity and inclusivity by supporting underrepresented groups through scholarships, mentorship, and professional development programmes, fostering a more equitable research environment.^4^

UNITE4TB (Academia and Industry United Innovation and Treatment for Tuberculosis) is a European Union Commission–funded public–private partnership involving academic institutions, small and medium-sized enterprises, public organisations, and pharmaceutical companies.^5^ Active from 2021 to 2028, the consortium spans across Europe, Asia, Africa, and South America and aims to deliver novel phase 2 clinical trials to accelerate the development of shorter, better-tolerated, highly effective TB treatment regimens.^6^ A key initiative of UNITE4TB is the Young Investigators Group (YIG), which focuses on empowering ECRs. Activities include participation in webinars in collaboration with the European Respiratory Society (ERS; https://www.ersnet.org/), drafting peer-reviewed papers, engaging with the Academy of the Tuberculosis Network European Trials group (TBnet; https://www.tbnet.eu/), and sharing ideas through online platforms. This work evaluates the impact of UNITE4TB on the professional development, mentorship, and capacity building of the ECRs involved in the YIG.

We conducted a cross-sectional assessment to evaluate consortium members’ and ECRs’ perspectives and experiences regarding research culture, mentoring, and the impact of consortium activities. Data collection involved two complementary approaches: a standardised online questionnaire and a live survey during the UNITE4TB annual meeting. The online questionnaire, developed using Google Forms, included single- and multiple-choice questions and Likert scale items. Participants were divided into two groups: 1) Consortium Members: Clinicians, researchers, and industry representatives, including senior investigators, project leaders, and mid-career researchers; 2) ECRs: Graduate students, postdoctoral fellows, and early-stage clinical researchers engaged in UNITE4TB activities. In addition to the online questionnaire, we conducted a live survey using the interactive presentation platform Mentimeter^7^ during the UNITE4TB annual meeting. This live poll aimed to gather real-time feedback from participants, focusing specifically on mentorship practices and the involvement of ECRs within the consortium. Data from the questionnaire and live survey were analysed to identify patterns, trends, and differences between the two participant groups. The survey was conducted between 29 April and 10 May 2024, targeting 243 consortium members and ECRs globally.

A total of 47 respondents (19.3%) completed the survey, of whom 26 (55.3%) were female and 36 (76.6%) were based in European countries. Most participants identified as researchers (57.4%), followed by clinicians (34%) and laboratory professionals (14.9%). Most respondents were affiliated with academia (68.1%), while equal proportions were from industry and independent consultancy (14.9%). Consortium members represented 66% of the respondents, with the remaining 34% being YIG members. Notably, only three participants were actively working at UNITE4TB trial sites.

Most consortium members who responded (74.2%) were aware of the YIG’s activities; however, only 16.1% reported active involvement, primarily through contributions to the UNITE4TB-ERS webinars. When asked about the benefits of having a YIG within UNITE4TB, members highlighted several key contributions. These included encouraging the participation of ECRs in clinical trials and TB research, enhancing their skills through real-world experiences, and fostering the development of well-trained leaders to ensure continuity in the field. Members also emphasised the YIG’s role in providing opportunities for learning, networking, and interdisciplinary collaborations, particularly benefiting ECRs from low- and middle-income countries (LMICs). Consortium members additionally identified advantages for the consortium itself. ECRs were seen as bringing fresh perspectives, innovative ideas, and energy that challenge traditional approaches. Their involvement fosters knowledge sharing, global connections, and collaborative learning across diverse settings and work packages. Moreover, engaging ECRs actively contributes to the long-term success and sustainability of the project by preparing the next generation of researchers who will drive future advancements in TB science. Despite these positive impacts, some consortium members noted a need for more straightforward communication regarding the YIG’s role and activities to enhance understanding and engagement.

Nonetheless, the YIG was widely recognised as a valuable asset for fostering innovation, capacity building, and sustainability in TB research, benefiting both ECRs and the consortium as a whole. Their primary motivations for joining included a strong interest in TB research (100%), opportunities for networking (75%), and the potential for career advancement (68.8%). Most respondents (87.5%) reported active involvement in YIG activities, particularly as discussants in joint UNITE4TB-ERS webinars and contributors to follow-up publications. Many appreciated the knowledge gained through webinars, publications, and discussions, significantly enhancing their clinical, diagnostic, and research skills. The YIG also provided insights, motivation, and connections with peers and senior researchers, fostering collaboration and a sense of community within the field. Mentorship emerged as a key factor, with respondents emphasising its role in broadening their professional horizons and equipping them with the tools to advance beyond their current positions.

During the UNITE4TB annual meeting, the survey results were presented. To involve the audience in the discussion, we conducted a live Mentimeter survey. Results are summarised in the Figure.

**Figure.**
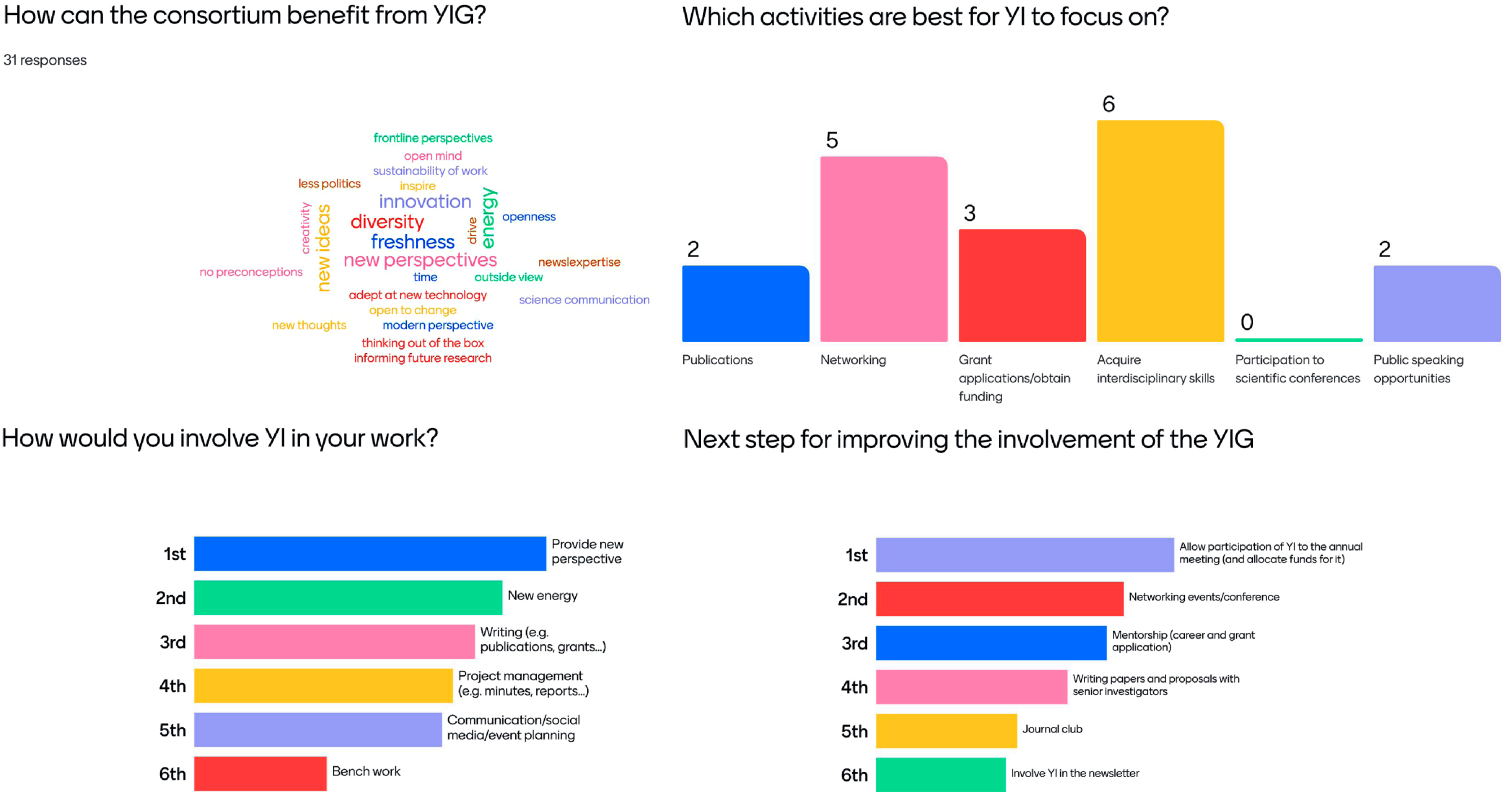
Results of the real-time online survey. YI = young investigators; YIG = Young Investigators Group.

Our survey provides valuable insights into the benefits of involving ECRs in large international collaborations. Mentorship emerged as a cornerstone for individual professional development and the advancement of the field.^8^ Both ECRs and consortium members highlighted the positive influence of the YIG within UNITE4TB, emphasising its role in fostering learning opportunities, networking, and leadership succession. Collaborative initiatives, such as organising webinars, journal clubs, and co-authoring grant proposals, were identified as key mechanisms to support ECRs and enhance their contributions.

An important finding of the study was the underrepresentation of ECRs from LMICs, a limitation that warrants attention given the global burden of TB. Mentorship is particularly valuable for ECRs in resource-limited settings, equipping them with the skills, confidence, and networks needed to navigate challenges such as limited funding, infrastructure, and opportunities. Strategies to enhance inclusivity involve leveraging virtual platforms and strengthening collaborations with institutions in LMICs, fostering connections and support without significant additional resources.^4,9,10^

To ensure the sustainability of the YIG, it is essential to integrate its activities within existing networks and foster collaborative efforts that extend beyond the lifespan of the UNITE4TB project. For example, ongoing partnerships with initiatives such as TBnet and ERS provide opportunities for shared learning, joint activities, and long-term engagement.^6,11–15^ Developing flexible formats, including virtual events and online resources, would also promote inclusivity and ensure continued participation. Scientific societies should invest in initiatives that go beyond traditional training by incorporating mentorship and networking to foster long-term career development for ECRs.

This study highlights the value of mentoring and engaging ECRs in international collaborations, not only as a means of fostering individual career development but also as a strategy for ensuring the continuity and diversity of TB research. Mentorship and structured programmes like the YIG empower the next generation of researchers to address global health challenges effectively, ensuring the field’s future success and sustainability.
